# Potassium-competitive acid blockers and advances in the management of patients with acid-related diseases: a narrative review

**DOI:** 10.3389/fphys.2025.1655102

**Published:** 2025-12-19

**Authors:** Xinwei Qiao, Peng Li

**Affiliations:** Department of Gastroenterology, Beijing Friendship Hospital, Capital Medical University, State Key Laboratory of Digestive Health, National Clinical Research Center for Digestive Disease, Beijing Key Laboratory of Early Gastrointestinal Cancer Medicine and Medical Devices, Beijing, China

**Keywords:** potassium-competitive acid blockers, gastroesophageal reflux disease, *Helicobacter pylori* eradication, peptic ulcer disease, narrative review

## Abstract

Traditional management of acid-related diseases, including gastroesophageal reflux disease, *Helicobacter pylori* infection, and peptic ulcer disease, primarily relies on proton pump inhibitors, which have notable limitations in acid suppression efficacy that hinder optimal treatment outcomes. Potassium-competitive acid blockers offer a novel approach with improved pharmacokinetic and pharmacodynamic properties, enhancing acid suppression and clinical efficacy in acid-related disease management. This narrative review evaluates the role of potassium-competitive acid blockers in acid-related disease management, comparing the pharmacokinetic, pharmacodynamic, clinical efficacy, and safety profiles of currently available agents. A comprehensive literature search from January 2002 to June 2024 was conducted across multiple databases to gather data on the efficacy and safety of potassium-competitive acid blockers. Potassium-competitive acid blockers showed comparable or superior clinical efficacy and generally comparable safety in various clinical settings vs. comparator proton pump inhibitors, particularly in managing erosive esophagitis, *H. pylori* eradication, and peptic ulcer disease. Potassium-competitive acid blockers, of which vonoprazan is the most well studied, offer comparable or improved therapeutic outcomes over traditional proton pump inhibitors, making them a valid option for several acid-related disease indications.

## 1 Introduction

Acid-related diseases (ARDs), which include gastroesophageal reflux disease (GERD), peptic ulcer disease (PUD), and *Helicobacter pylori* infections, continue to be a major healthcare concern despite advances in management strategies (Inatomi et al., 2016). ARD management primarily involves gastric acid suppression, with current options including proton pump inhibitors (PPIs) that covalently and irreversibly bind to H^+^-K^+^-ATPase (Oshima and Miwa, 2018). PPIs have been available for more than 30 years and are considered the cornerstone treatment for patients with ARDs (Turshudzhyan et al., 2022). However, PPIs: 1) only bind to active proton pumps, 2) have a short half-life, 3) are influenced by food intake, 4) have a relatively slow onset action requiring 3–5 days of dosing for the optimal acid suppression, and 5) show high interindividual variability in pharmacokinetics (PK) due to genetic polymorphism of hepatic cytochrome P4502C19 (CYP2C19) (Sachs et al., 2007; Otake et al., 2016; Buzas and Birinyi, 2023; Katzka and Kahrilas, 2023). These limitations contribute to the inability of PPIs to maintain a prolonged increased intragastric pH critical for mucosal healing in patients with erosive esophagitis (EE) and the eradication of *H. pylori* (Buzas and Birinyi, 2023; Scarpignato et al., 2023). In the management of ARDs, the percentage of time intragastric pH is above 4 (pH > 4 holding time ratio [HTR]) should be more than 75% within 24 h for healing EE, while prolonged pH 6 HTR >75% is required for optimum *H. pylori* eradication since bacterial replication is active. Antibiotic effectiveness is optimized in this environment (Scarpignato et al., 2023; Yaozong, 2023). Based on published literature, PPIs have a 24-h intragastric pH 4 HTR of up to 65.1% with once-daily dosing and pH 6 HTR of up to 69% with twice-daily dosing at day 7, which may not be adequate in achieving the standard pH HTR required for EE healing and *H. pylori* eradication (Sakurai et al., 2015a; Kagami and Furuta, 2016).

Meanwhile, potassium-competitive acid blockers (P-CABs) bind to the K^+^ site of H^+^/K^+^ ATPase to block access of the K^+^ ion to the proton pump to prevent H^+^ secretion; they form non-covalent and reversible bonds with H^+^-K^+^-ATPase and competitively bind to both active and inactive proton pumps in a K^+^-competitive manner (Inatomi et al., 2016; Otake et al., 2016). Unlike PPIs, P-CABs have different structures compared with PPIs, are acid stable, and do not require enteric coating. P-CABs are also not dependent on food intake, are not prodrugs, and have long half-lives. These properties result in more rapid attainment of peak plasma levels with potent and consistent onset of acid suppression, irrespective of CYP2C19, compared with PPIs (Inatomi et al., 2016; Sugano, 2018; Leowattana and Leowattana, 2022; Wong et al., 2022; Buzas and Birinyi, 2023).

Six P-CABs are currently approved and available in clinical practice for different indications: revaprazan, vonoprazan, tegoprazan, fexuprazan, keverprazan, and zastaprazan. Meanwhile, clinical trials are ongoing for X842 (Leowattana and Leowattana, 2022; Blair, 2024; Scarpignato and Hunt, 2024). Each of these P-CABs has different pharmaceutical structures as well as PK and pharmacodynamic (PD) characteristics that may translate to different clinical properties. This narrative review provides an overview of these currently available P-CABs for managing patients with ARD. The structure, PK and PD properties, clinical efficacy, and safety of P-CABs in patients with ARD are also compared, to guide daily clinical practice.

## 2 Search strategy

This narrative review involved a comprehensive search of available publications on P-CABs in ARDs from 1 January 2002 to 15 June 2024 using PubMed, Embase, and clinical trial registration websites, as well as press releases and product labeling, and summarized key PK and PD properties, clinical efficacy, and safety profile to provide a reference for clinical practice. Study selection was limited to articles in English involving human subjects. The following filters were applied: Clinical Study, Clinical Trial, Meta-analysis, Systematic Review, Randomized Clinical Trial, and Practice Guideline.

To ensure comprehensive coverage, detailed search terms for drug names, disease conditions, and related interventions were used. See the Supplementary Appendix for the full list of search terms used.

## 3 Pharmacokinetics and pharmacodynamics of P-CABs

**FIGURE 1:**
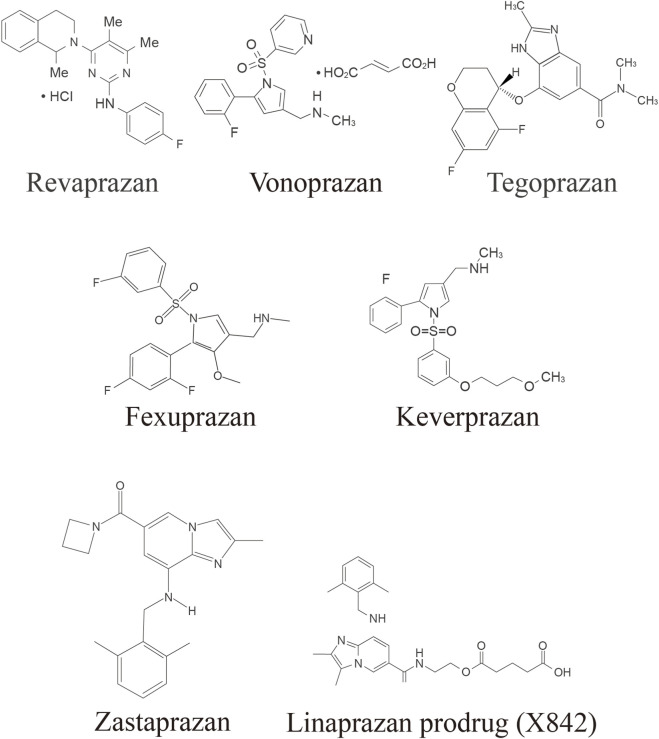
Structures of currently available P-CABs (Leowattana and Leowattana, 2022; Blair, 2024; Scarpignato and Hunt, 2024). P-CAB, potassium-competitive acid blocker.

An overview of the differences in structures and PK and PD properties of currently available P-CABs are provided in Figure 1 and Supplementary Table S1.

First, revaprazan has a pyrimidine structure and PK properties of plasma peak (T_max_) at 1.3–2.5 h after a single dose of up to 200 mg, with a mean elimination time (T_1/2_) of 2.2–2.4 h (Leowattana and Leowattana, 2022). Regarding PD, the 24-h intragastric pH 4 HTR for revaprazan at 200 mg was 25.1% on day 1 after a single dose and 25.3% on day 7 after multiple doses (Sunwoo et al., 2020). Although it can rapidly inhibit gastric acid secretion, revaprazan has only been able to increase intragastric pH up to 5 (Oshima and Miwa, 2018).

Vonoprazan has a pyrrole structure and lacks the imidazopyridine ring common in other P-CABs. It has a high negative log of the acid dissociation constant (pKa) of 9.3, which makes it a basic compound that remains un-ionized in the acidic canaliculi of the gastric parietal cells and is expected to bind to proton pumps regardless of acid secretion. Its median T_max_ ranges from 1.5 to 2.0 h for 10–40 mg once daily (QD). It has a mean T_1/2_ of up to 9 h, which means that vonoprazan can stay in the blood longer and block acid secretion continuously (Jenkins et al., 2015). In terms of PD, vonoprazan 20 mg QD also has a rapid and sustained acid-inhibitory effect, with means 24-h intragastric pH 4 HTRs of 84.2% on day 1 and 93.8% on day 7 (Sakurai et al., 2015a). Studies have also shown that the 24-h intragastric pH 6 HTRs for vonoprazan 20 mg QD and 20 mg twice daily (BID) were 74% and 85%, respectively (Kagami and Furuta, 2016). These properties collectively contribute to vonoprazan’s improved potency and durability of acid suppression compared to PPIs and other P-CABs (Supplementary Table S1) (Jenkins et al., 2015; Srinivas, 2016).

Fexuprazan also has a pyrrole structure like vonoprazan (Leowattana and Leowattana, 2022). It has a T_max_ of 1.75–3.5 h with single doses of 10–320 mg and a T_1/2_ of 9.1 h with the standard 40 mg dose. At 40 mg QD, fexuprazan has 24-h intragastric pH 4 HTRs of 44.6% on day 1 and 55.7% on day 7 (Sunwoo et al., 2018; Daewoong, 2022).

Tegoprazan has a benzimidazole structure and a pKa of 5.1, T_max_ of 1.84 h (0.83–3.00 h) after a single dose of 50 mg, and T_1/2_ of about 4.1 h (Han et al., 2023; Scarpignato and Hunt, 2024). The 24-h intragastric pH 4 HTR of tegoprazan 50 mg QD was 58.55% (Han et al., 2023).

Keverprazan has an imidazopyridine structure, a T_max_ of 1.25–1.75 h, and T_1/2_ ranging from 6.00 to 7.17 h from 5 to 60 mg QD (Zhou et al., 2023b). At 20 mg QD, keverprazan had 24-h intragastric pH 4 HTRs on days 1 and 7 of 85.0% and 98.3%, respectively (Zhou et al., 2023a).

Zastaprazan also has an imidazopyridine structure. At 5–60 mg, zastaprazan has a T_max_ of 0.5–1.5 h and T_1/2_ of 6–9 h. Zastaprazan 20 and 40 mg had 24-h intragastric pH 4 HTRs of 85% and 92%, respectively (Blair, 2024).

## 4 Clinical evidence for P-CABs and acid-related diseases

### 4.1 Gastroesophageal reflux disease

#### 4.1.1 Erosive esophagitis

##### 4.1.1.1 Vonoprazan efficacy

In a randomized, double-blind, phase 3 study in Japan, 409 patients with EE (Los Angeles [LA] Classification Grades A–D) received either vonoprazan 20 mg or lansoprazole 30 mg QD. Results showed that the EE healing rate was non-inferior between the two groups at 8 weeks. The healing rates of the vonoprazan and lansoprazole groups were 96.6% vs. 92.5% at 4 weeks (difference: 4.1%; 95% confidence interval [CI], −0.31–8.55; p < 0.0001 for non-inferiority) and 99.0% vs. 95.5% at 8 weeks (difference: 3.5%; 95% CI, 0.36–6.73; p < 0.0001 for non-inferiority), respectively. Importantly, the healing rate in severe EE (LA grade C/D) was significantly greater in the vonoprazan vs. lansoprazole group (98.7% vs. 87.5%; p = 0.0082) (Ashida et al., 2016). Another phase 3 study that included patients with EE from China, South Korea, Taiwan, and Malaysia also showed the non-inferior efficacy of vonoprazan vs. lansoprazole in terms of EE healing rates at 8 weeks (92.4% vs. 91.3%; difference: 1.1%; 95% CI, −3.82–6.09). Similar findings were also observed in favor of vonoprazan regarding healing rate in severe EE (LA grade C/D) (Xiao et al., 2020).

A randomized phase 3 study in the United States also investigated the healing rates of EE in 1,024 adults who were assigned to receive either vonoprazan 20 mg QD or lansoprazole 30 mg QD for up to 8 weeks. This was followed by a rerandomization to vonoprazan 10 or 20 mg QD or lansoprazole 15 mg QD for 24 weeks in those patients with healing. In terms of healing rates at 8 weeks, vonoprazan was non-inferior and superior to lansoprazole (92.9% vs. 84.6%; difference in healing rates: 8.3%; 95% CI, 4.5–12.2; p < 0.0001 for non-inferiority and superiority). Secondary analyses also showed that vonoprazan was superior in healing LA grade C/D esophagitis at week 2 (difference in healing rates: 17.6%; 95% CI, 7.4–27.4; p = 0.0008 for superiority). Similar results in favor of vonoprazan 10 or 20 mg QD vs. lansoprazole 15 mg QD were noted in terms of the maintenance of EE healing in 878 patients, including in those with LA grade C/D esophagitis (Laine et al., 2023).

In the maintenance phase, a randomized phase 3 study also showed the non-inferiority and superiority of vonoprazan 10 and 20 mg QD vs. lansoprazole 15 mg QD in EE recurrence rate after a 24-week maintenance in Japan (EE recurrence rates at 24 weeks: 5.1%, 2.0%, and 16.8%, respectively; p < 0.0001 for non-inferiority for both vonoprazan 10 and 20 mg vs. lansoprazole 15 mg, p = 0.0002 for superiority of vonoprazan 10 mg vs. lansoprazole 15 mg, and p < 0.0001 for superiority of vonoprazan 20 mg vs. lansoprazole 15 mg) (Ashida et al., 2018). Similarly, a double-blind, multicenter, phase 3 clinical trial with patients from China, South Korea, and Malaysia demonstrated non-inferiority and superiority in EE recurrence rate after a 24-week maintenance therapy with vonoprazan at 10 and 20 mg QD (13.3% and 12.3%) vs. lansoprazole 15 mg QD (25.5%) (differences in EE recurrence rates with vonoprazan 10 and 20 mg vs. lansoprazole 15 mg: −12.3%; 95% CI, −20.3 to −4.3; −13.3%; 95% CI, −21.3 to −5.3) (Xiao et al., 2024).

##### 4.1.1.2 Tegoprazan efficacy

In a randomized phase 3 study from South Korea, tegoprazan at 50 or 100 mg QD was non-inferior to esomeprazole 40 mg QD in achieving cumulative healing rates in 302 patients with EE at week 8 (per-protocol set [PPS], 98.9% across all treatment groups; differences in EE healing rates with 50 and 100 mg tegoprazan vs. esomeprazole 40 mg: −0.1%; 95% CI, −3.0–3.1; 0.0%; 95% CI, −3.0–3.1; p < 0.0001 for non-inferiority in both comparisons) (Lee et al., 2019). In another phase 3 study from South Korea that included patients with predominantly mild EE (LA grade A: 57.3%, LA grade B: 37.3%; N = 351), tegoprazan 25 mg was demonstrated to be non-inferior to lansoprazole 15 mg in maintaining mild EE healing (endoscopic remission rates of 90.6% vs. 89.5%, respectively, after 24 weeks; difference: 1.1%; 95% CI, −6.2–8.3; p = 0.0014) (Cho et al., 2023).

##### 4.1.1.3 Fexuprazan efficacy

A phase 3 trial in South Korea included 263 adults with endoscopically confirmed EE (LA grades A–D) and received either fexuprazan 40 mg QD or esomeprazole 40 mg QD. At week 8, fexuprazan was non-inferior to esomeprazole in terms of cumulative healing rates (PPS, 99.1% in both treatment groups; common risk difference of healing rates: 0.89%; 95% CI, −0.86–2.64) (Lee et al., 2022).

Similar results were reported in a phase 3 study in China. Fexuprazan 40 mg QD was non-inferior to esomeprazole 40 mg QD in terms of mucosal healing rates at 8 weeks, i.e., full analysis set (FAS) results of 88.5% vs. 89.0% at 8 weeks, respectively (PPS, 97.3% vs. 97.9%, respectively) (Zhuang et al., 2024).

##### 4.1.1.4 Keverprazan efficacy

A multicenter, phase 3 clinical trial in China involving 238 patients with EE demonstrated that healing rates at 8 weeks with keverprazan 20 mg QD were non-inferior to lansoprazole 30 mg QD (FAS, 95.8% vs. 89.9%; difference: 5.8%; 95% CI, −0.6–12.3; p = 0.081). Keverprazan treatment was also associated with numerically higher EE healing rates vs. lansoprazole in patients with severe EE (i.e., LA grade C/D; FAS, 91.7% vs. 80.0%, p = 0.417; PPS, 95.2% vs. 78.3%; p = 0.188) (Chen et al., 2022).

##### 4.1.1.5 Zastaprazan efficacy

A phase 3 study in South Korea showed that mucosal healing rate with zastaprazan 20 mg QD was non-inferior to esomeprazole 40 mg QD at week 8 for the treatment of EE in 300 patients with predominantly low-grade EE (FAS, 97.92% vs. 94.93%, respectively; p = 0.178) (Oh et al., 2025).

#### 4.1.2 Non-erosive reflux disease

##### 4.1.2.1 Vonoprazan efficacy

A randomized clinical trial in the United States included 458 patients with presumed non-erosive reflux disease (NERD) who responded symptomatically to vonoprazan 20 mg QD during a 4-week run-in period. A total of 207 patients then continued to receive on-demand treatment with vonoprazan 10, 20, or 40 mg, or placebo QD for another 6 weeks. Results showed that significantly more patients who received on-demand vonoprazan regardless of the dose vs. placebo achieved complete heartburn relief within 3 h of on-demand dosing, and this was sustained for 24 h (10-mg group, 56.0%; 20-mg group, 60.6%; 40-mg group, 70.0%; placebo group, 27.3%; p < 0.0001 vs. placebo for each vonoprazan group). Heartburn resolution was also significantly greater with vonoprazan vs. placebo within 1 h of on-demand use (10 mg, 35.9%; 40 mg, 26.6%; placebo, 18.1%; p < 0.0001 and p < 0.01 vs. placebo comparisons, respectively) (Fass et al., 2023).

##### 4.1.2.2 Tegoprazan efficacy

In a phase 3 trial in South Korea, 324 patients with NERD received either tegoprazan 50 or 100 mg QD or placebo for 4 weeks. Results showed significantly higher rates of complete resolution of heartburn and regurgitation with both tegoprazan groups vs. placebo (42.5% and 48.5% vs. 24.2%; differences in rates of complete resolution of heartburn with tegoprazan 50 or 100 mg vs. placebo: 18.3% and 24.3%; p = 0.0058 and p = 0.0004, respectively). Both tegoprazan groups were also associated with significantly higher complete-resolution rates of heartburn and proportions of heartburn-free days vs. placebo (p < 0.05 for all) (Kim et al., 2021).

#### 4.1.3 Summary on P-CAB efficacy in GERD

P-CABs, most notably vonoprazan and tegoprazan, demonstrate robust non-inferiority or superiority to PPIs for initial and maintenance therapy in GERD, particularly for severe disease. Comparable results with comparator PPIs were also reported for fexuprazan, keverprazan, and zastaprazan, supporting the expanding role of P-CABs in GERD management. A detailed summary of key study details, treatment interventions, and efficacy endpoints and results is provided in Supplementary Table S2.

### 4.2 *Helicobacter pylori* eradication

#### 4.2.1 Vonoprazan efficacy

The efficacy of vonoprazan-based quadruple therapy has been demonstrated in a phase 3 study involving 455 patients with treatment-naïve and previously treated *H. pylori* infections in Asia (i.e., China, South Korea, and Taiwan). Patients positive for *H. pylori* infection received bismuth-containing quadruple therapy (BQT; i.e., amoxicillin 1 g BID, clarithromycin 500 mg BID, and bismuth potassium citrate/bismuth tripotassium dicitrate 600 mg BID) plus either vonoprazan 20 mg BID or lansoprazole 30 mg BID, for 2 weeks, followed by vonoprazan or lansoprazole monotherapy QD (4 weeks maximum). Results showed that the eradication rate with vonoprazan-based BQT was 91.5%, which was non-inferior to that of lansoprazole-based BQT (86.8%; difference: 4.7%, 95% CI, −1.28–10.69). Importantly, the addition of vonoprazan exceeded the clinically meaningful threshold of *H. pylori* eradication (90%) (Hou et al., 2022).

Similarly, a phase 3 study from China showed that vonoprazan-based BQT was non-inferior to esomeprazole-based BQT in achieving *H. pylori* eradication in 510 patients (i.e., eradication rates in FAS at week 4 post treatment, 86.8% vs. 86.7%, respectively; difference: 0.1%; 95% CI, −5.95–6.17; PPS, 87.4% vs. 86.3%, respectively; difference: 1.2%; 95% CI, −5.03–7.36) (Song et al., 2022).

For triple therapy *H. pylori* eradication strategies, a randomized, double-blind, multicenter, parallel-group study in Japan recruited 650 *H. pylori*-positive patients who received either vonoprazan 20 mg BID or lansoprazole 30 mg BID in combination with BID amoxicillin 750 mg and clarithromycin 200 or 400 mg for 7 days as first-line triple therapy. The first 50 patients who failed first-line therapy then received second-line vonoprazan-based triple therapy (i.e., vonoprazan 20 mg, amoxicillin 750 mg, and metronidazole 250 mg, all BID, as open-label treatment). Results showed that the eradication rate in the vonoprazan group was non-inferior and numerically higher vs. the lansoprazole group (92.6% vs. 75.9%; difference: 16.7%; 95% CI, 11.2–22.1; p < 0.0001). Second-line vonoprazan-based triple therapy also resulted in a high eradication rate (98.0%; 95% CI, 89.4–99.9). The eradication rate was also significantly higher with vonoprazan vs. lansoprazole in those patients infected with clarithromycin-resistant strains (82.0% vs. 40.0%; p < 0.0001) (Murakami et al., 2016).

In the United States and Europe, the approval of vonoprazan-based triple and dual therapies for *H. pylori* eradication was based on efficacy and safety data from the phase 3, randomized PHALCON-HP trial involving 1,046 patients. This trial demonstrated that vonoprazan-based triple (vonoprazan 20 mg plus amoxicillin 1 g and clarithromycin 500 mg, BID) and dual therapies (vonoprazan 20 mg BID plus amoxicillin 1 g thrice daily [TID]) for 14 days were superior to lansoprazole-based triple therapy (lansoprazole 30 mg plus amoxicillin 1 g and clarithromycin 500 mg, BID) in the eradication rates for all patients (80.8% and 77.2%, respectively, vs. 68.5%, differences: 12.3%; 95% CI, 5.7–18.8; p < 0.001; 8.7%; 95% CI, 1.9–15.4; p = 0.013) (Chey et al., 2022).

As dual therapy regimens, a prospective randomized controlled study validated that a 14-day combination of vonoprazan 20 mg BID and amoxicillin 750 mg four times daily (QID) as first-line eradication therapy was superior to esomeprazole-based BQT (esomeprazole 20 mg, amoxicillin 1 g, clarithromycin 500 mg, colloidal bismuth subcitrate 220 mg, BID) in the intention-to-treat (ITT) population (N = 316; ITT analysis: 89.9% and 81.0%, respectively; difference, ITT: 8.9%; 95% CI, 1.2–16.5; p = 0.037; PPS, 97.9% and 90.8%, respectively; difference: 7.2%; 95% CI, 1.8–12.4; p = 0.009) (Peng et al., 2023).

An open-label randomized controlled study in China was the first to evaluate and demonstrate that first-line dual therapy with vonoprazan 20 mg BID plus tetracycline 500 mg TID was non-inferior to PPI-based BQT (i.e., lansoprazole 30 mg BID plus colloidal bismuth 150 mg, tetracycline 500 mg, and metronidazole 400 mg, TID) in the treatment of *H. pylori* infection in patients with penicillin allergy (N = 300). The eradication rates in the vonoprazan group and the lansoprazole group were as follows: ITT analysis, 92.0% and 89.3%; difference: 2.7%; 95% CI, −4.6–10.0; p = 0.000 for non-inferiority; PPS, 95.1% and 97.7%; difference: 2.6%; 95% CI, −2.9–8.3; p = 0.000 for non-inferiority) (Gao et al., 2024).

#### 4.2.2 Tegoprazan efficacy

In a multicenter, randomized, double-blind, double-dummy phase 3 clinical study in China that included 561 treatment-naïve patients with *H. pylori* infection, FAS results showed that tegoprazan-based BQT (50 mg BID) was superior to esomeprazole-based BQT (20 mg BID). The eradication rates of tegoprazan-based BQT and esomeprazole-based BQT were 93.5% and 86.4%, respectively (difference: 7%; 95% CI, 2.06–11.99; p = 0.0055 for non-inferiority and superiority) (Zhou and Song, 2024).

A randomized, double-blind study in South Korea showed that tegoprazan-based BQT (i.e., tegoprazan 50 mg and placebo BID, tetracycline 500 mg QID, metronidazole 500 mg TID, and bismuth subcitrate 300 mg QID, for 14 days) was non-inferior to lansoprazole-based BQT (i.e., lansoprazole 30 mg and placebo BID, tetracycline 500 mg QID, metronidazole 500 mg TID, and bismuth subcitrate 300 mg QID, for 14 days) for *H. pylori* eradication. In 211 patients with *H. pylori* infection, eradication rates for the tegoprazan and lansoprazole groups in the ITT population were 80.0% and 77.4%, respectively (difference: 2.6%; 95% CI, −8.4–13.7; p = 0.0124 for non-inferiority) (Kim et al., 2023).

#### 4.2.3 Keverprazan efficacy

With respect to quadruple therapy, a phase 3 trial reported that keverprazan 20 mg was non-inferior to esomeprazole 20 mg in *H. pylori* eradication when added to clarithromycin 500 mg, amoxicillin 1 mg, and bismuth potassium citrate 240 mg, all taken BID for 2 weeks. FAS results showed the *H. pylori* eradication rates were 87.8% and 82.52% for the keverprazan and esomeprazole groups, respectively (difference: 5.29%; 95% CI, −0.55–11.18; p = 0.0750). In the PPS, keverprazan was superior to esomeprazole (93.49% vs. 88.24%, respectively; difference: 5.25%; 95% CI, 0.29–10.45; p = 0.0382 for superiority) (Tan et al., 2024).

#### 4.2.4 Summary of P-CAB efficacy in *H. pylori* eradication

Regimens based on P-CABs, especially vonoprazan and tegoprazan, achieved clinically meaningful eradication rates, including among clarithromycin-resistant strains, with efficacy profiles comparable to or exceeding established PPIs. Keverprazan-based BQT also demonstrated comparable results with a PPI-based BQT in *H. pylori* eradication. A summary of the study regimens and *H. pylori* eradication rate outcomes for these P-CABs is detailed in Supplementary Table S2.

### 4.3 Peptic ulcer disease

#### 4.3.1 Gastric and duodenal ulcers

##### 4.3.1.1 Vonoprazan efficacy

Two phase 3, non-inferiority, randomized, multicenter studies in Japan reported that in 482 patients, the endoscopically confirmed healing rates of gastric ulcers were 93.5% for vonoprazan 20 mg QD at 8 weeks and 93.8% for lansoprazole 30 mg QD in the FAS population (difference: −0.3%; 95% CI, −4.75–4.21; p = 0.0011 for non-inferiority). Although the non-inferiority of vonoprazan vs. lansoprazole for duodenal ulcer healing was not confirmed in the FAS, it was confirmed in the secondary analysis of the PPS (i.e., proportions of healed patients at week 6, 97.1% vs. 98.9%, respectively; difference: −1.8%; 95% CI, −4.70–1.19; p = 0.0171) (Miwa et al., 2017). The aforementioned randomized phase 3 study on Asian patients also reported that vonoprazan 20 mg QD was non-inferior to lansoprazole 30 mg QD for duodenal ulcer healing in patients with *H. pylori* infection (96.9% vs. 96.5%, respectively, 6 weeks treatment; difference: 0.4%; 95% CI, −3.00–3.79) (Hou et al., 2022).

##### 4.3.1.2 Tegoprazan efficacy

A randomized, phase 3 study from South Korea included 306 patients diagnosed with gastric ulcers who were assigned to receive either tegoprazan 50 or 100 mg or lansoprazole 30 mg QD for 4 or 8 weeks. In the FAS, cumulative healing rates confirmed by endoscopy at 8 weeks for tegoprazan 50 and 100 mg and lansoprazole 30 mg were 94.8%, 95.0%, and 95.7%, respectively (95% CI for cumulative healing rates with tegoprazan 50 mg vs. lansoprazole 30 mg, −7.98–6.09; p = 0.0177 for non-inferiority; 95% CI for cumulative healing rates with tegoprazan 100 mg vs. lansoprazole 30 mg, −7.69–6.31; p = 0.0146 for non-inferiority). Corresponding healing rates for the PPS were 100.0%, 97.9%, and 100.0%, respectively. Similar healing rates were evident across all treatment groups as early as 4 weeks (FAS, 90.6%, 91.9%, and 89.2%, respectively; p-values for tegoprazan 50 and 100 mg vs. lansoprazole 30 mg were p = 0.0117 and p = 0.0040, respectively). These results show that ulcer healing rates with both tegoprazan doses were non-inferior to lansoprazole at 4 and 8 weeks (Cho et al., 2020).

##### 4.3.1.3 Revaprazan efficacy

In a randomized phase 3 study in South Korea, 292 patients with gastric ulcers received either revaprazan 200 mg or omeprazole 20 mg to compare cumulative healing rates after 4 and 8 weeks of treatment. Results demonstrated that revaprazan and omeprazole had comparable cumulative recovery percentages in the ITT population (week 4: 82.5% vs. 79.9%, p = 0.565; week 8: 93.0% vs. 89.6%, difference: 3.4%; 95% CI, −3.1–9.9; p = 0.304). The PPS also noted similar results at 8 weeks, i.e., healing rates of 99.1% vs.100%, respectively (difference: −0.9%; 95% CI, −2.4–0.6; p = 0.3229) (Chang et al., 2007).

For duodenal ulcers, a randomized, double-blind phase 3 study in South Korea compared the efficacy of revaprazan 200 mg QD vs. omeprazole 20 mg QD in terms of achieving complete ulcer healing by endoscopy at 4 weeks in 228 patients. Healing rates for revaprazan and omeprazole were similar in both the ITT population (i.e., 91.7% and 91.3%, respectively; difference: 0.4%; 95% CI, −7.0–7.7; p = 0.9228) and PPS (i.e., 94.4% and 92.3%, respectively; difference: 2.1%; 95% CI, −4.9–9.1; p = 0.5666) (Chung et al., 2005).

##### 4.3.1.4 Keverprazan efficacy

A randomized, double-blind phase 3 study in China demonstrated the efficacy profile of keverprazan in patients with duodenal ulcers. Among the 360 patients included in the study, healed duodenal ulcer rates at week 6 in the keverprazan and lansoprazole groups were 94.4% and 93.3%, respectively (difference: 1.2%; 95% CI, −4.0%–6.5%; p = 0.6404), suggesting that keverprazan 20 mg QD was non-inferior to lansoprazole 30 mg QD for duodenal ulcer healing over 6 weeks (Tan et al., 2023).

#### 4.3.2 Low-dose aspirin- and non-steroidal anti-inflammatory drug–induced peptic ulcer disease

##### 4.3.2.1 Vonoprazan efficacy

In a phase 3 study, 621 patients with low-dose aspirin (LDA)–induced PUD were randomly assigned to receive either vonoprazan (10 or 20 mg QD) or lansoprazole (15 mg QD). Results showed that vonoprazan 10  and 20 mg were non-inferior to lansoprazole 15 mg in preventing PUD recurrence after 24 weeks of treatment (recurrence rates, 0.5%, 1.5%, and 2.8%, respectively; Farrington and Manning test: margin 8.7%, significance level 2.5%). Post hoc analyses of the extension study (≤2 years) showed that, compared with lansoprazole 15 mg, PUD recurrence rates were significantly lower with vonoprazan 10 mg (p = 0.039) but not vonoprazan 20 mg (p = 0.260). Gastrointestinal bleeding rates were higher with lansoprazole vs. either dose of vonoprazan in both the 24-week and extension periods of the study (post hoc analysis: vonoprazan 10 mg vs. lansoprazole 15 mg, p = 0.018; vonoprazan 20 mg vs. lansoprazole 15 mg, p = 0.019) (Kawai et al., 2018).

Similarly, a phase 3 study enrolled 640 patients with a history of PUD and requiring long-term non-steroidal anti-inflammatory drug (NSAID) therapy. Results confirmed the non-inferiority of vonoprazan to lansoprazole in terms of recurrence prevention of NSAID-induced PUD at 24 weeks (3.3%, 3.4%, and 5.5% for vonoprazan 10 or 20 mg and lansoprazole 15 mg, respectively; differences in PUD recurrence rates with vonoprazan 10 and 20 mg vs. lansoprazole 15 mg: −2.2%; 95% CI, −6.18–1.83; −2.1%; 95% CI, −6.13–1.97; p < 0.001 for both vonoprazan doses vs. lansoprazole) (Mizokami et al., 2018).

#### 4.3.3 Summary on P-CAB efficacy in PUD

Vonoprazan, tegoprazan, revaprazan and keverprazan are at least non-inferior to PPIs in-terms of gastric and duodenal ulcer healing. Vonoprazan, in particular, may be useful for secondary prevention of NSAID-induced PUD, owing to PUD recurrence rates comparable to or lower than those of lansoprazole. See Supplementary Table S2 for a summary of the key efficacy outcomes of the discussed P-CABs in PUD.

### 4.4 Safety summary of P-CABs

In general, the mentioned studies on P-CABs demonstrated comparable tolerability and safety profiles between P-CABs and corresponding PPI controls in the initial and maintenance therapy for both newly diagnosed and recurring EE (Ashida et al., 2016; Ashida et al., 2018; Lee et al., 2019; Xiao et al., 2020; Chen et al., 2022; Lee et al., 2022; Cho et al., 2023; Laine et al., 2023; Xiao et al., 2024; Zhuang et al., 2024; Oh et al., 2025). Increased gastrin level was a common adverse event associated with vonoprazan, fexuprazan, keverprazan, tegoprazan, and zastaprazan. In particular, several clinical trials reported that vonoprazan was consistently associated with greater or more frequent, dose-dependent increases in gastrin levels vs. lansoprazole (Ashida et al., 2016; Murakami et al., 2016; Miwa et al., 2017; Ashida et al., 2018; Kawai et al., 2018; Mizokami et al., 2018; Cho et al., 2020; Xiao et al., 2020; Chen et al., 2022; Hou et al., 2022; Lee et al., 2022; Fass et al., 2023; Laine et al., 2023; Tan et al., 2023; Xiao et al., 2024; Zhou and Song, 2024; Oh et al., 2025). Similar findings were reported in clinical trials comparing keverprazan with lansoprazole, and zastaprazan with esomeprazole (Tan et al., 2023; Oh et al., 2025). However, the observed serum gastrin increases across the P-CABs reviewed were not associated with clinically significant effects on the gastric mucosa based on histopathologic examinations. Levels also returned to baseline levels after treatment discontinuation.

In the *H. pylori* eradication studies, P-CAB–based regimens generally had comparable safety profiles to PPI-based regimens (Murakami et al., 2016; Chey et al., 2022; Hou et al., 2022; Kim et al., 2023; Tan et al., 2024; Zhou and Song, 2024). Importantly, dual therapy showed better safety profiles vs. PPI-based quadruple therapy. In the dual regimen study containing vonoprazan, the incidence of adverse events was significantly lower vs. the esomeprazole-based BQT group (19.0% vs. 43.0%; p < 0.001) (Peng et al., 2023).

Meanwhile, similar safety profiles between P-CABs and the corresponding PPI controls were reported across the different studies on gastric and duodenal ulcer healing and in LDA-induced PUD (Chung et al., 2005; Chang et al., 2007; Miwa et al., 2017; Kawai et al., 2018; Mizokami et al., 2018; Cho et al., 2020; Hou et al., 2022; Tan et al., 2023).

## 5 Discussion

P-CABs provide a viable alternative to PPIs in the management of patients with ARD by overcoming the limitations associated with PPIs (Inatomi et al., 2016; Katzka and Kahrilas, 2023). In particular, the superior PK and PD properties of P-CABs with respect to acid suppression—rapid and potent acid suppression, minimal interindividual variability in acid control, and sustained efficacy (i.e., prolonged HTRs at higher pH levels) after a single dose vs. PPIs—potentially make them a preferable option for ARD management (Sakurai et al., 2015b; Otake et al., 2016; Sugano, 2018; Buzas and Birinyi, 2023; Katzka and Kahrilas, 2023; Scarpignato et al., 2023). As demonstrated in the reviewed clinical studies (Supplementary Table S2), P-CABs have comparable or superior clinical efficacy and generally comparable safety as PPIs across different ARD indications. In particular, vonoprazan appears as the most established P-CAB due to its robust clinical trial evidence, and it is currently acknowledged by several treatment guidelines for the treatment of GERD (Jung et al., 2021; Xiao et al., 2021; Iwakiri et al., 2022) and *H. pylori* eradication (as a component of quadruple-, triple-, and dual-therapy regimens) (Kato et al., 2019; Malfertheiner et al., 2022; Zhou et al., 2022; Chey et al., 2024), and PUD (Kamada et al., 2021). Meanwhile, preliminary PK and PD studies indicate that the newer P-CABs keverprazan and zastaprazan have also achieved acid suppression levels comparable to vonoprazan, suggesting strong potency across this drug class (Zhou et al., 2023a; Blair, 2024). Nevertheless, it should be emphasized that in this review, all other available P-CABs, including fexuprazan, keverprazan, revaprazan, tegoprazan, and zastaprazan, have demonstrated at least non-inferiority to PPIs in randomized clinical trials across various ARDs.

To appreciate the efficacy of P-CABs in ARD management and, in particular, the potential clinical advantages of vonoprazan vs. other P-CABs, it is important to identify the key differences in the PK and PD properties between vonoprazan and other available P-CABs, which are summarized in Supplementary Table S1.

In GERD, achieving mucosal healing is related to the degree and duration of acid suppression and the length of treatment, which may also help achieve effective symptomatic control that may be challenging with current PPIs (Scarpignato and Hunt, 2024). Although PPIs are considered first-line treatment for GERD, first-line P-CABs—specifically, vonoprazan and tegoprazan—have been recognized as valid options due to their demonstrated efficacy and safety in GERD clinical trials (Jung et al., 2021; Xiao et al., 2021; Iwakiri et al., 2022). For instance, treatment with vonoprazan achieved up to 99.0% healing rate for patients with EE at 8 weeks and up to 98.7% for those with severe EE (LA grades C/D), whereas tegoprazan treatment achieved healing rates of up to 98.9% (Ashida et al., 2016; Lee et al., 2019).

The consistent efficacy associated with vonoprazan in EE may be attributed to its more potent and sustained acid suppression vs. other P-CABs. Initial 20 mg administration of vonoprazan exceeds the ideal 24-h intragastric pH 4 HTR threshold of >75% required for mucosal healing, especially in severe EE cases (i.e., LA grade C/D) (Supplementary Table S1) (Sakurai et al., 2015a; Zhang et al., 2022; Buzas and Birinyi, 2023; Yaozong, 2023). Across multiple phase 3 trials, vonoprazan achieved higher and faster healing rates and lower recurrence rates than PPI comparators such as lansoprazole (Ashida et al., 2016; Ashida et al., 2018; Xiao et al., 2020; Laine et al., 2023; Xiao et al., 2024). Other P-CABs have also demonstrated non-inferiority for EE healing and maintenance vs. standard PPIs, although data for severe EE are limited.

Sustained intragastric pH > 6 enhances antibiotic efficacy in *H. pylori* eradication, a target often unmet by PPIs but consistently reached with vonoprazan. (Malfertheiner et al., 2022; Zhang et al., 2022; Buzas and Birinyi, 2023; Scarpignato et al., 2023; Yaozong, 2023; Scarpignato and Hunt, 2024). This PD advantage is reflected in clinical trials showing that vonoprazan-based regimens consistently resulted in higher eradication rates vs. PPI-based therapy, including in clarithromycin-resistant infections (Murakami et al., 2016; Chey et al., 2022; Song et al., 2022; Peng et al., 2023). Emerging studies of other P-CABs also suggest non-inferiority for eradication compared to PPIs (Supplementary Table S2).

Published 24-h intragastric pH 6 HTRs for other P-CABs are limited, and among those with available values, only vonoprazan at the standard 20 mg BID regimen for *H. pylori* eradication achieves the ideal 24-h intragastric pH 6 HTR >75% (Supplementary Table S1) (Kagami and Furuta, 2016; Yaozong, 2023). In a more recent multicenter randomized trial, vonoprazan 20 mg BID plus amoxicillin 1 g TID dual therapy achieved a median pH of 7.4 on day 1 and 7.8 on day 7, with dynamic 24-h profiles showing gastric pH > 6 within 4–5 h of first dosing and maintained at pH 7 to 9 thereafter (Song et al., 2025). These findings reinforce the potent and sustained acid suppression of vonoprazan beyond earlier reports. It is also associated with prolonged nocturnal acid suppression, i.e., nocturnal pH 4 HTR of 84.3% after one dose of vonoprazan 20 mg QD (Sakurai et al., 2015a). These properties suggest that vonoprazan may have advantages over existing acid-suppressing drugs in providing early response due to the maximum efficacy after the first dose, which may help ensure increased antibiotic stability and efficacy and concomitant high *H. pylori* eradication rates (Jenkins et al., 2015; Scarpignato et al., 2023; Song et al., 2025).

Comparable *H. pylori* eradication rates were also reported for tegoprazan-based quadruple therapy and keverprazan-based quadruple therapy vs. PPI-based comparator groups (Kim et al., 2023; Tan et al., 2024; Zhou and Song, 2024). These results suggest that the benefits of P-CABs in the context of *H. pylori* eradication may extend to other agents in this class. However, further studies are needed to establish whether their PD properties of acid suppression vs. PPIs consistently translate into clinical efficacy in treating *H. pylori* infections.

Vonoprazan, tegoprazan, revaprazan, and keverprazan have generally shown comparable efficacy and safety vs. PPIs in treating gastric and duodenal ulcers (Chung et al., 2005; Chang et al., 2007; Miwa et al., 2017; Cho et al., 2020; Hou et al., 2022; Tan et al., 2023). Vonoprazan, in particular, demonstrated non-inferior healing vs. PPIs for gastric and duodenal ulcers, achieving healing rates of up to 93.8% at 8 weeks and 97.1% at 6 weeks (Miwa et al., 2017). Vonoprazan also had non-inferior (10 mg QD) and superior results (20 mg QD) vs. lansoprazole in the secondary prevention of LDA-induced PUD (Kawai et al., 2018). Tegoprazan treatment also achieved non-inferiority to lansoprazole for gastric ulcer healing, achieving up to approximately 95% healing rate at 8 weeks (Cho et al., 2020).

Collectively, these efficacy data have led to including P-CABs in multiple regional and international guidelines for GERD, *H. pylori* eradication, and PUD (Kato et al., 2019; Jung et al., 2021; Kamada et al., 2021; Xiao et al., 2021; Iwakiri et al., 2022; Malfertheiner et al., 2022; Zhou et al., 2022; Association. et al., 2023; Chey et al., 2024).

While the clinical efficacy of P-CABs has been demonstrated across regions, it is important to note that their approval and routine clinical use remain geographically limited. Notably, the recently published Mexican consensus guidelines for the management of GERD are among the first in Latin America to recommend P-CABs alongside PPIs as drugs of choice for both EE and NERD, as well as for patients with refractory symptoms. This inclusion reflects the growing recognition of P-CABs’ clinical value in new regions, even as regulatory approval in many countries is still pending (Valdovinos Diaz et al., 2024).

Alongside regulatory and clinical considerations, drug cost may represent a major barrier to the wider adoption of P-CABs. For instance, in North America, these agents are typically priced higher than established generic PPIs and may face inconsistent insurance coverage (pmarketresearch.com, 2024). Meanwhile, P-CABs have been demonstrated to be more cost-effective than PPIs based on several cost-effectiveness analyses in Asia (Wang et al., 2022; Miwa et al., 2023). While the health economic implications and cost-effectiveness of P-CABs vs. existing therapies are important issues, a detailed evaluation is beyond the scope of this review.

While our review includes studies published through June 2024, new clinical data on additional P-CABs are emerging. For example, a recent randomized trial in Korea established the non-inferiority of on-demand tegoprazan vs. esomeprazole for GERD (Kang et al., 2025). Results from ongoing phase 3 trials are also expected to support the efficacy of tegoprazan in patients with EE and NERD (NCT05587309, NCT05587322) (ClinicalTrials.gov, 2025a; ClinicalTrials.gov, 2025b). Similarly, prospective data also now support the efficacy and safety of fexuprazan in patients with laryngopharyngeal reflux disease (Kim et al., 2024). These findings attest to the expanding role of P-CABs as a class for managing diverse ARDs.

Overall, the safety profiles of P-CABs are broadly similar and overlap with those of PPIs across the different studies and ARD indications in this narrative review. No new safety signals were reported, and patients generally tolerated all P-CABs well. While some earlier P-CABs, such as the imidazopyridine compound SCH28080 and its derivative, linaprazan, were withdrawn due to hepatotoxicity, this adverse event was not reported in any of the clinical studies on the newer agents reviewed here (Oshima and Miwa, 2018).

*Clostridium difficile* infection (CDI) is a recognized complication associated with prolonged and potent gastric acid suppression, as previously observed with PPIs and, more recently, with P-CABs such as vonoprazan (Otake et al., 2016; Oshima and Miwa, 2018; Sugano, 2018; Wong et al., 2022). Although CDI was not reported in the P-CAB clinical studies included in this review, available evidence and mechanistic studies suggest that both PPIs and vonoprazan may similarly increase CDI risk, particularly with longer treatment duration or higher acid suppression (Mori and Suzuki, 2019; Saruta et al., 2023; Ouyang et al., 2024). Further studies are warranted to clarify the association between chronic vonoprazan and other P-CAB use and CDI.

Hypergastrinemia is a concern with prolonged acid suppression, which has been associated with gastric endocrine hyperplasia and increased risk of carcinoid tumor development with chronic PPI use, although long-term consequences remain unclear (Sugano, 2018). The increase in serum gastrin concentration is believed to be a consequence of the potent gastric acid antisecretory effect of PPIs, which was also demonstrated in the clinical studies on P-CABs included in this review (Murakami et al., 2016; Miwa et al., 2017; Sugano, 2018; Lee et al., 2022; Wong et al., 2022; Fass et al., 2023; Laine et al., 2023; Oh et al., 2025). Among the P-CABs reviewed, vonoprazan was notably associated with consistently greater increases in serum gastrin than with lansoprazole treatment, which may be due to its comparatively more potent gastric acid suppression vs. other P-CABs demonstrated in PK and PD studies (Sakurai et al., 2015a; Ashida et al., 2016; Inatomi et al., 2016; Kagami and Furuta, 2016; Ashida et al., 2018). Keverprazan and zastaprazan were the only other P-CABs demonstrated to have greater serum gastrin increases vs. their comparator PPIs, consistent with their comparable pH 4 HTRs with vonoprazan (Tan et al., 2023; Zhou et al., 2023a; Blair, 2024; Oh et al., 2025).

Despite the associated hypergastrinemia observed with P-CAB treatment, no adverse clinical effects or significant gastric mucosal changes were reported in the clinical trials reviewed. Gastrin levels also consistently returned to baseline after cessation of therapy (Murakami et al., 2016; Miwa et al., 2017). Notably, even with extended vonoprazan administration of up to 104 weeks in the trial on LDA-associated PUD prevention, no gastric endocrine cell tumors were reported, despite associated increases in gastrin levels (Kawai et al., 2018). This was further supported by the results of the 5-year VISION study in patients with EE. Findings revealed that while maintenance therapy with vonoprazan 10 mg QD was associated with higher gastrin, parietal cell hyperplasia, and foveolar hyperplasia than maintenance lansoprazole 10 mg QD, no increased risk of malignant epithelial alterations or gastric neuroendocrine tumors was observed. The VISION trial is the only study to date providing 5-year safety data for a P-CAB (Uemura et al., 2018; Uemura et al., 2025). Nevertheless, further long-term safety data remain essential as more potent P-CABs and broader indications emerge (Wong et al., 2022; Scarpignato and Hunt, 2024).

### 5.1 Conclusions

P-CABs overcome several pharmacologic limitations of PPIs that translate to clinically comparable or superior therapeutic efficacy and safety in GERD, *H. pylori* eradication, and PUD. Among the currently available P-CABs, vonoprazan has the most well-established PK and PD profile, and it has predictably become the standard P-CAB investigated in different clinical trials on ARD due to its potent and durable acid suppression. Its proven efficacy and safety have been recognized by clinical guidelines and consensus recommendations across several ARD indications. However, establishing the long-term safety of P-CABs warrants further research.
