# Supplementary material for: Potassium-competitive acid blockers and advances in the management of patients with acid-related diseases: a narrative review
Source: Front Physiol. 2025 Dec 19;16:1655102. doi: 10.3389/fphys.2025.1655102 (PMC12757290; doi:10.3389/fphys.2025.1655102)
Supplement: Supplementary file 1 [file Supplementaryfile1.docx]

**Appendix**

**Search terms and filters used**

- Date range: 1 January 2002 – 15 June 2024
- Language: English
- Population: Human

***Filters applied:***

- Clinical Study
- Clinical Trial
- Randomized Clinical Trial
- Meta-analysis
- Systematic Review
- Practice Guideline
- Human
- English

***Drug search terms:***

- "Vonoprazan" OR "TAK 438" OR "TAK438" OR "TAK-438"
- "Tegoprazan" OR full chemical nomenclature variants
- "Revaprazan" OR "YH-1885" OR full chemical nomenclature variants
- "Keverprazan"
- "Fexuprazan" OR "DWP14012" OR "DWP-14012" OR full chemical nomenclature variants OR "abeprazan"
- "Zastaprazan" OR "JP-1336"

***Disease search terms:***

*GERD:*

- "Reflux esophagitis", "Esophagitides, Peptic", "Peptic Esophagitis", "Esophagitis, Reflux", "Reflux Esophagitis", "RE", "erosive esophagitis", "EE"
- "Non erosive reflux disease", "NERD", various nomenclature variants

*PUD:*

- "Stomach Ulcers", "Ulcer, Stomach", "Gastric Ulcer", "GU"
- "Duodenal ulcer", "DU", "Curling Ulcer", various nomenclature variants
- "Peptic Ulcer", "Gastroduodenal Ulcer", "Marginal Ulcer", various nomenclature variants

*Interventions Related to PUD:*

- AND ("LDA" OR "aspirin" OR "Low-dose aspirin")
- AND ("NSAID" OR all relevant nomenclature variants)

*Helicobacter pylori Infection:*

- ("Helicobacter pylori" OR nomenclature variants) AND "Infection"
